# Modulation of RASD2 by miRNA‐485‐5p Drives Thyroid Cancer Progression and Metastasis

**DOI:** 10.1002/kjm2.70028

**Published:** 2025-04-28

**Authors:** Xiao‐Yu Li, Jian‐Ping Sun, Hao Guo, Xiao‐Qing Fan, Shan‐Shan Zhang, Bo Wang, Na Yu, Qing‐Huai Li

**Affiliations:** ^1^ Department of Thyroid and Breast Surgery The Second Hospital of Hebei Medical University Shijiazhuang China; ^2^ Department of Neurosurgery The Second Hospital of Hebei Medical University Shijiazhuang China

**Keywords:** miRNA‐485‐5p, papillary thyroid cancer, RASD2

## Abstract

This study investigates the role of RASD2 (Ras Homolog Enriched In Striatum) in thyroid carcinoma progression and its modulation by microRNA‐485‐5p. Differential RASD2 expression patterns were initially identified through bioinformatic analysis of public databases. Immunohistochemical staining and quantitative reverse transcription PCR (qRT‐PCR) validated these findings in clinical specimens and cell lines. Functional characterization of RASD2 was performed through loss‐of‐function studies, examining cellular proliferation, invasion, and glycolytic parameters. The prognostic significance of RASD2 was evaluated through Kaplan–Meier analysis. Using integrated bioinformatic approaches, we identified miRNA‐485‐5p as a potential RASD2 regulator and confirmed this interaction through molecular studies. The therapeutic potential of targeting RASD2 was assessed using xenograft and pulmonary metastasis models. RASD2 showed significant upregulation in thyroid cancer tissues, with elevated expression correlating with adverse clinicopathological parameters including lymphatic metastasis, extrathyroidal invasion, and advanced TNM stage. Genetic silencing of RASD2 in IHH4 and TPC‐1 cells substantially impaired their malignant phenotypes, manifesting as decreased proliferation, invasion, and glycolytic activity. Mechanistically, we identified miRNA‐485‐5p as a crucial negative regulator of RASD2, whose overexpression recapitulated the tumor‐suppressive effects of RASD2 knockdown. In vivo studies further validated the therapeutic potential of RASD2 inhibition, demonstrating reduced tumor growth and metastatic burden. Our findings establish the miRNA‐485‐5p/RASD2 axis as a critical regulatory pathway in thyroid cancer progression, offering new insights into disease pathogenesis and potential therapeutic interventions.

## 1 Introduction

Thyroid cancer encompasses diverse malignancies arising from thyroid gland cells [1]. These neoplasms originate primarily from follicular and parafollicular cells, manifesting as distinct clinical entities [1, 2]. Among these, papillary thyroid carcinoma (PTC) represents the predominant form, followed by follicular thyroid carcinoma (FTC), while anaplastic thyroid carcinoma (ATC), though rare, exhibits the most aggressive clinical course [1, 2]. The heterogeneity of thyroid malignancies reflects the complex cellular architecture of the gland, with each subtype displaying unique molecular signatures and biological behaviors [2, 3]. Recent decades have witnessed a significant surge in thyroid cancer incidence, partly attributed to advanced diagnostic technologies and improved surveillance methods [3, 4]. Despite the generally favorable outcomes with surgical management in early‐stage disease, patients with advanced or metastatic thyroid cancer face poor prognosis. This therapeutic challenge underscores the critical need to decipher the molecular mechanisms driving thyroid cancer progression and metastasis.

Ras Homolog Enriched In Striatum (RASD2), a member of the Ras superfamily of small GTPases, was initially characterized by its abundant expression in striatal tissues and its involvement in neurological disorders, including epilepsy and depression [5, 6]. Recent evidence has expanded RASD2's biological significance beyond neurological contexts, revealing its crucial role in oncogenesis. Studies have demonstrated that elevated RASD2 expression correlates with unfavorable clinical outcomes in gastrointestinal stromal tumors and gliomas [5, 7]. Furthermore, in uveal melanoma, RASD2 has emerged as a key oncogenic mediator, promoting cellular proliferation and metastatic dissemination [8]. However, the functional significance of RASD2 in thyroid cancer pathogenesis remains largely unexplored, warranting comprehensive investigation.

MicroRNAs (miRNAs), small non‐coding RNA molecules, serve as crucial post‐transcriptional regulators of gene expression [9]. In thyroid cancer, several miRNAs, including miR‐146b, miR‐221, miR‐206, and miR‐4428, have emerged as significant modulators of disease progression and clinical outcomes [10, 11]. Through computational analysis using miRNA prediction algorithms, we identified RASD2 as a potential target of miR‐485‐5p. This miRNA has demonstrated tumor‐suppressive properties across multiple cancer types, including oral cavity carcinoma, breast cancer, and gastrointestinal malignancies [12, 13, 14, 15]. Despite its established role in other cancers, the functional significance of miR‐485‐5p in thyroid carcinogenesis remains to be elucidated.

In this study, we investigated the functional role of RASD2 and its regulation by miR‐485‐5p in thyroid cancer through comprehensive analyses of clinical specimens, cell lines, and animal models. Our findings revealed that RASD2 promotes thyroid cancer progression through enhanced cellular proliferation, invasion, and glycolytic metabolism, while miR‐485‐5p acts as a tumor suppressor by directly targeting RASD2. These results not only expand our understanding of thyroid cancer pathogenesis but also suggest that targeting the miR‐485‐5p/RASD2 axis may represent a promising therapeutic strategy for thyroid cancer treatment.

## 2 Materials and Methods

### 2.1 Clinical Specimen Collection

Clinical specimens comprising 96 matched pairs of thyroid cancer and adjacent normal tissues were obtained from patients undergoing surgical treatment at The Second Hospital of Hebei Medical University. All patients included in this study underwent surgical resection of primary thyroid cancer at our institution between January 2018 and December 2022. The inclusion criteria were: (1) pathologically confirmed primary thyroid cancer; (2) no prior chemotherapy, radiotherapy, or targeted therapy; (3) complete clinical and follow‐up data available; and (4) availability of both tumor and paired adjacent normal tissue samples. Patients were excluded if they met any of the following criteria: (1) history of previous thyroid surgery; (2) presence of other concurrent malignancies; (3) severe systemic diseases or organ dysfunction; (4) pregnancy or lactation; and (5) incomplete clinical information or tissue samples unsuitable for analysis. The basic clinical characteristics of all included patients were summarized in Table 1, stratified by RASD2 expression levels.

**TABLE 1:** Correlation between the expression level of RASD2 and clinicopathological features in PTC patients.

Clinical characters	Cases (%)	RASD2 expression		*p* *
		High	Low	
	*n* = 96	(*n* = 48)	(*n* = 48)	
Age (years)				
≤ 45	55 (57.29)	25	30	0.409
> 45	41 (42.71)	23	18	
Gender				
Male	22 (22.92)	10	12	0.809
Female	74 (77.08)	38	36	
Tumor size (cm)				
≤ 2	82 (85.42)	39	43	0.386
> 2	14 (14.58)	9	5	
**Extrathyroidal extension**				
**No**	**80 (83.33)**	**35**	**45**	**0.012**
**Yes**	**16 (16.67)**	**13**	**3**	
**TNM Stage**				
**I + II**	**70 (72.92)**	**30**	**40**	**0.038**
**III + IV**	**26 (27.08)**	**18**	**8**	
**Lymph node metastasis**				
**N0**	**45 (46.88)**	**17**	**28**	**0.040**
**N1a + N1b**	**51 (53.12)**	**31**	**20**	

*Note*: Significant results were in bold.

* Chi‐Square detection.

Fresh tissue samples (approximately 1 cm [3]) were snap‐frozen in liquid nitrogen and maintained at −80°C until RNA extraction and subsequent RT‐qPCR analysis of RASD2 expression. The study protocol adhered to the Declaration of Helsinki guidelines (Code of Ethics, AF‐SOP‐07‐1.1‐01) and received approval from the Ethics Committee of The Second Hospital of Hebei Medical University. All participants provided written informed consent before enrollment. Thyroid cancer diagnosis was independently confirmed through histological examination by three experienced pathologists, with tumor classification and staging performed according to World Health Organization (WHO) criteria.

### 2.2 Data Mining and Bioinformatic Analysis

Differential gene expression analysis was conducted using the Gene Expression Omnibus (GEO) database (https://www.ncbi.nlm.nih.gov). Dataset selection was restricted to “Homo sapiens” studies containing “papillary thyroid cancer” samples. The GEO2R analytical tool was employed to identify differentially expressed genes, with statistical significance defined as *p* < 0.05 and absolute fold change |FC| > 1. RASD2 expression patterns in thyroid cancer were analyzed using the GEPIA platform, while miR‐485‐5p expression was examined through the TCGA database using UALCAN.

### 2.3 Prognostic Evaluation

For survival analysis, patients were stratified into high and low RASD2 expression groups using the median RT‐qPCR expression value as the cutoff threshold. Associations between RASD2 expression levels and clinicopathological features were evaluated using chi‐square tests. Kaplan–Meier survival curves were constructed to assess prognostic differences between groups, and survival analysis was performed using logistic regression.

### 2.4 Immunoblotting

Total protein was extracted from tissue samples using RIPA lysis buffer supplemented with protease inhibitor cocktail (Roche, #04693159001). After centrifugation (12,000 × *g*, 15 min, 4°C), protein concentrations were determined using the Pierce BCA Protein Assay Kit (Thermo Scientific, #23225). Protein samples (10 μg) were denatured at 95°C for 10 min and separated by SDS‐PAGE on 10% gels. Following transfer to PVDF membranes, blots were blocked with 5% non‐fat milk and incubated with primary antibodies against RASD2 (1:1000, Abcam, ab67277) and *β*‐actin (1:5000, Cell Signaling #4970) overnight at 4°C. After washing with TBST, membranes were incubated with HRP‐conjugated anti‐rabbit IgG (1:5000, Cell Signaling #7074) for 1 h at room temperature. Protein bands were visualized using enhanced chemiluminescence substrate (Pierce ECL Western Blotting Substrate, Thermo Scientific #32106). The intensity of protein bands was quantified using ImageJ software (version 1.53, National Institutes of Health, USA), with values normalized to the corresponding actin band and expressed as fold change relative to control.

### 2.5 Cell Culture

Five thyroid cell lines were used in this study: Nthy‐ori 3‐1 (normal thyroid follicular epithelial cells) and four thyroid cancer cell lines (BCPAP, IHH4, KTC, and TPC‐1). All cell lines were obtained from the American Type Culture Collection (ATCC, Manassas, VA, USA). Cells were maintained in RPMI‐1640 medium supplemented with 10% fetal bovine serum (FBS) and 1% penicillin–streptomycin at 37°C in a humidified incubator with 5% CO_2_. Cells were passaged when reaching 80%–90% confluence using 0.25% trypsin–EDTA solution.

### 2.6 Stable RASD2 Knockdown Cell Line Generation

Short hairpin RNA (shRNA) targeting RASD2 and scrambled control shRNA were cloned into the pLKO.1‐puro lentiviral vector (Addgene, #8453). The sequences were shRASD2 5′‐CTTCCACCGTAAGGTATACAA‐3′, and scrambled control shRNA 5′‐ACGTTCGACATAACATACGCT‐3′. Lentiviral particles were produced in HEK293T cells by co‐transfecting the shRNA‐expressing plasmid with packaging plasmids psPAX2 (Addgene, #12260) and envelope plasmid pMD2.G (Addgene, #12259) using Lipofectamine 3000 (Invitrogen). Viral supernatants were collected 48 and 72 h post‐transfection, filtered through 0.45 μm filters, and concentrated using PEG‐it Virus Precipitation Solution (System Biosciences). Target cells were infected with lentiviral particles in the presence of 8 μg/mL polybrene. After 48 h, stable cell lines were selected using 1.5 μg/mL puromycin for 2 weeks.

### 2.7 Transient Transfection

The full‐length human RASD2 cDNA was amplified and cloned into pcDNA3.1(+) vector (Genescript). The construct was verified by DNA sequencing. miR‐485‐5p mimic, miR‐485‐5p inhibitor, and their corresponding negative controls were purchased from MedChemExpress (MCE, NJ, USA). Cells were seeded in 6‐well plates and grown to 70%–80% confluence before transfection. Transfection was performed using Lipofectamine 3000 (Invitrogen) according to the manufacturer's instructions. The final concentrations were 2 μg/well for plasmid DNA and 50 nM for miRNA mimic/inhibitor. Cells were harvested 48 h post‐transfection for subsequent experiments.

### 2.8 qRT‐PCR Analysis

Total RNA was extracted using TRIzol reagent (Takara, Japan) and treated with DNase I. First‐strand cDNA was synthesized from 1 μg total RNA using the PrimeScript RT reagent Kit (Takara) according to the manufacturer's protocol. Quantitative PCR was performed using SYBR Premix Ex Taq II (Takara) on an ABI 7500 Fast Real‐Time PCR System (Applied Biosystems). The following primers were used: RASD2 Forward: 5′‐CATCCTCACAGGAGATGTCTTCAT‐3′, Reverse: 5′‐TTTTTATTCTTCAGGCAGGACTTGA‐3′; *β*‐actin Forward: 5′‐CATGTACGTTGCTATCCAGGC‐3′, and Reverse: 5′‐CTCCTTAATGTCACGCACGAT‐3′; miR‐485‐5p Forward: 5′‐CGAGAGGCTGGCCGTGAT‐3′, Reverse: 5′‐AGTGCAGGGTCCGAGGTATT‐3′; U6 Forward: 5′‐GCTTCGGCAGCACATATACTAAAAT‐3′, and Reverse: 5′‐CGCTTCACGAATTTGCGTGTCAT‐3′. Each 20 μL reaction contained 2 μL cDNA (250 ng/μL), 10 μL SYBR Green master mix, 0.5 μL each of forward and reverse primers (10 μM), and 7 μL nuclease‐free water. PCR conditions were: 94°C for 2 min, followed by 40 cycles of 94°C for 10 s, 60°C for 15 s, and 72°C for 30 s. Melting curve analysis was performed to confirm reaction specificity. Relative expression levels were calculated using the 2^−ΔΔCT^ method, with *β*‐actin as the internal control for RASD2 and U6 as the control for miRNA.

### 2.9 Cell Proliferation Assays

Cell proliferation was assessed using two complementary methods. For the CCK‐8 assay, cells were seeded at 3000 cells per well in 96‐well plates. At designated time points (0, 24, 48, and 72 h), 10 μL of CCK‐8 solution (Dojindo) was added to each well and incubated for 3 h at 37°C. Absorbance was measured at 450 nm using a microplate reader.

For EdU incorporation assay, cells were seeded on glass coverslips in 24‐well plates. The Click‐iT EdU Cell Proliferation Kit (Thermo Fisher Scientific) was used according to the manufacturer's instructions. Briefly, cells were incubated with 10 μM EdU for 2 h, fixed with 4% paraformaldehyde for 15 min, and permeabilized with 0.5% Triton X‐100 for 20 min at room temperature. After washing with 3% BSA in PBS, cells were incubated with Click‐iT reaction cocktail for 30 min at room temperature in the dark. Nuclei were counterstained with DAPI (1:1000). Images were captured using a fluorescence microscope (Olympus) and the percentage of EdU‐positive cells was calculated from at least five random fields.

### 2.10 Glycolytic Flux Quantification

Cells were seeded in 96‐well plates (2 × 10^4^ cells/well) and cultured for 24 h. Glucose consumption was measured in glucose‐supplemented media using the glucose oxidase method with a Glucose Assay Kit (Cat# K686‐100, BioVision, USA). Lactate production was quantified using the Lactate Assay Kit (Cat# K607‐100, BioVision, USA) based on lactate dehydrogenase detection. Intracellular ATP levels were determined using the CellTiter‐Glo Luminescent Cell Viability Assay (Cat# G7570, Promega, USA). All measurements were performed according to the manufacturer's protocols and normalized to cell number. Data were collected using a microplate reader (absorbance at 450 nm for glucose/lactate, luminescence for ATP) and expressed relative to control conditions.

### 2.11 miRNA‐Target Interaction Analysis

Potential miRNA regulators of RASD2 were identified using three independent bioinformatic platforms: miRDB, StarBase, and TargetScan. The overlapping predictions identified hsa‐miR‐485‐5p as a candidate regulator of RASD2. The predicted interaction was validated using dual‐luciferase reporter assays. The wild‐type RASD2 3′UTR sequence containing the predicted miR‐485‐5p binding site was amplified and cloned into the pmirGLO dual‐luciferase vector (Promega) between XhoI and XbaI restriction sites. The mutant construct was generated by site‐directed mutagenesis of the miR‐485‐5p binding sequence. Cells were seeded in 24‐well plates (5 × 10^4^ cells/well) and co‐transfected with either wild‐type or mutant reporter constructs (500 ng) along with miR‐485‐5p mimic or negative control (50 nM). After 48 h, luciferase activities were measured using the Dual‐Luciferase Reporter Assay System (HY‐K1013, MedChemExpress). Renilla luciferase activity was normalized to firefly luciferase activity. Results were expressed as relative light units compared to control conditions.

### 2.12 Invasive Capacity Evaluation

Cell invasion was assessed using Transwell chambers (8 μm pore size, Corning #3422) coated with Matrigel (BD Biosciences #356234, diluted 1:8 in serum‐free medium). The Matrigel layer was allowed to polymerize at 37°C for 2 h prior to cell seeding. Cells (5 × 10^4^ in 250 μL serum‐free medium) were seeded in the upper chamber, while the lower chamber contained 750 μL complete growth medium (with 10% FBS) as a chemoattractant. After 24‐h incubation at 37°C in 5% CO_2_, non‐invading cells were removed from the upper surface of the membrane using a cotton swab. Invaded cells on the lower surface were fixed with 4% paraformaldehyde for 15 min and stained with 0.1% crystal violet for 15 min. Five random fields per insert were photographed under a light microscope (×200 magnification) and quantified using ImageJ software.

### 2.13 In Vivo Tumorigenesis and Metastasis Analysis

Animal studies were conducted following approval from the Animal Ethics Committee of The Second Hospital of Hebei Medical University and complied with the Institutional Animal Welfare Guidelines. For the subcutaneous xenograft model, TPC‐1 cells stably expressing sh‐RASD2 or sh‐NC (1 × 10^6^ cells in 100 μL PBS:Matrigel, 1:1) were injected into the right flank of 4‐week‐old male BALB/c nude mice (18–20 g, Beijing Vital River Laboratory Animal Technology Co. Ltd.). Mice were randomly assigned to experimental groups (*n* = 5 per group) using a computer‐generated sequence. Tumor dimensions were measured weekly using digital calipers, and volume was calculated as (length × width^2^)/2. For the experimental metastasis model, 5 × 10^5^ cells in 100 μL PBS were injected via the lateral tail vein (*n* = 5 per group). Animals were monitored daily and humanely euthanized by CO_2_ inhalation after 35 days (subcutaneous model) or 28 days (metastasis model). Tumors and lungs were harvested, weighed, photographed, and fixed in 10% neutral buffered formalin for histopathological analysis. Metastatic foci in the lungs were counted under a microscope following H&E staining. All experiments were performed in accordance with ARRIVE guidelines, with investigators blinded to group allocation during data collection and analysis.

### 2.14 Histological and Immunohistochemical Analyses

Tissue specimens were fixed in Bouin's solution for 24 h, dehydrated through a graded ethanol series, embedded in paraffin, and sectioned at 5 μm thickness. For histological analysis, sections were deparaffinized in xylene, rehydrated through graded alcohols, and stained with a hematoxylin and eosin kit (H&E, Abcam). For immunohistochemistry, antigen retrieval was performed in 10 mM sodium citrate buffer (pH 6.0) using microwave heating (95°C, 15 min). Endogenous peroxidase activity was quenched with 3% H_2_O_2_ for 10 min. Sections were blocked with 10% normal rabbit serum for 30 min and incubated with primary antibodies against RASD2 (1:200, Abcam, ab67277) or Ki‐67 (1:100, Abcam, ab16667) overnight at 4°C. After PBS washes, sections were incubated with HRP‐conjugated secondary antibody (1:500, Vector Laboratories) for 45 min at 37°C, followed by visualization using a DAB substrate kit (Vector Laboratories). Sections were counterstained with hematoxylin, dehydrated, and mounted. Images were captured using a light microscope (Olympus BX53) and analyzed using ImageJ software. Five random fields per section were quantified by investigators blinded to the experimental groups.

### 2.15 Statistical Methodology

Statistical analyses were performed using GraphPad Prism version 8.0 (GraphPad Software, San Diego, CA). Data are presented as mean ± standard deviation (SD) from three independent experiments. Normal distribution was assessed using the Shapiro–Wilk test. Comparisons between two groups were analyzed using a two‐tailed unpaired Student's *t*‐test, while one‐way ANOVA followed by Tukey's post hoc test was used for multiple group comparisons. Correlation analyses were performed using Pearson's correlation coefficient. Statistical significance was set at *p* < 0.05, with significance levels indicated as *p < 0.05, **p < 0.01, and ***p < 0.001.

## 3 Results

### 3.1 RASD2 Is Upregulated in Thyroid Cancer and Associates With Poor Prognosis

**FIGURE 1:**
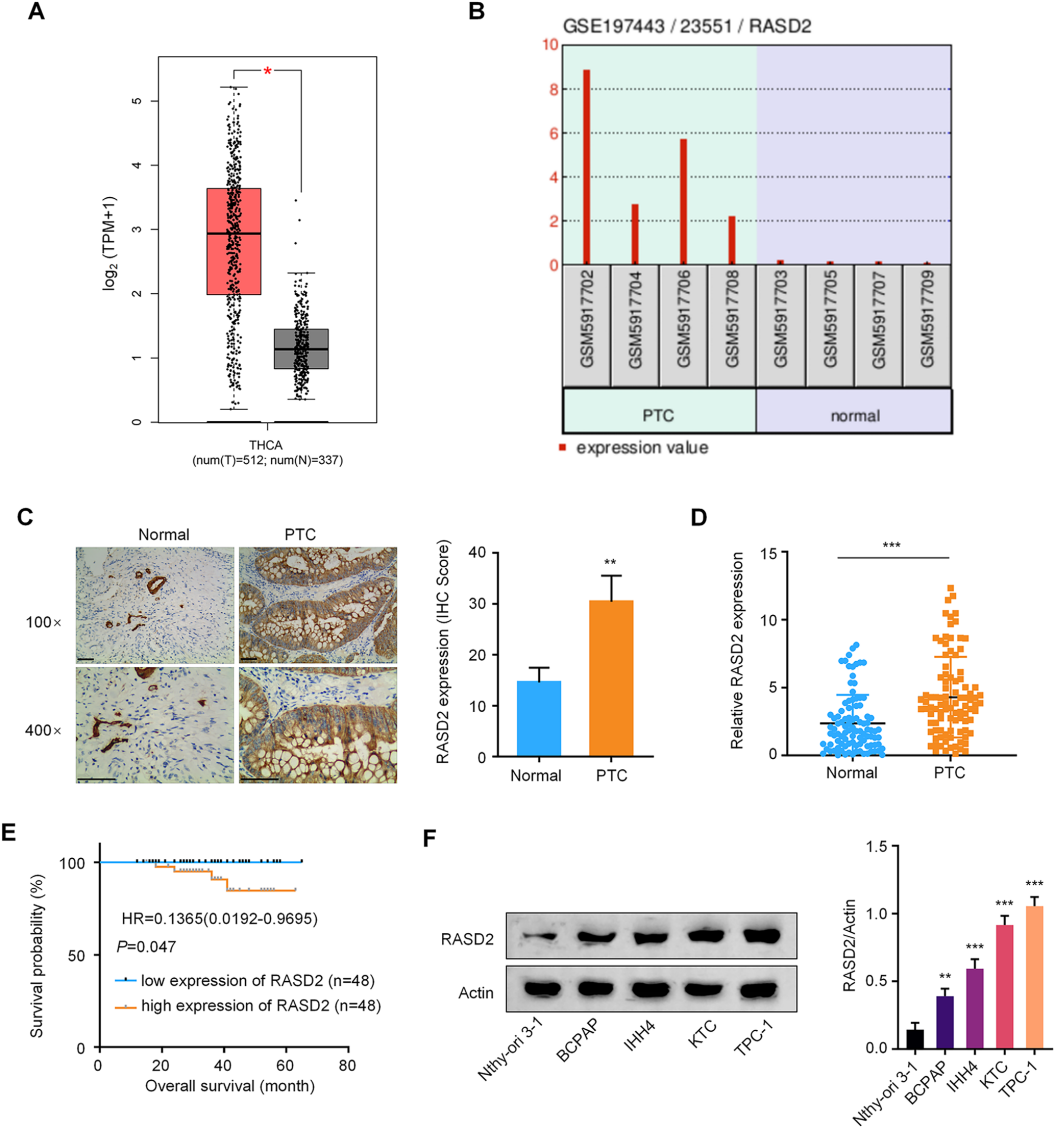
RASD2 expression analysis in thyroid cancer. (A) GEPIA database analysis comparing RASD2 expression between thyroid cancer and normal thyroid tissues. (B) RASD2 expression analysis using GEO dataset (GSE197443) in matched tumor‐normal pairs. (C) Representative immunohistochemical staining of RASD2 in paired thyroid cancer and adjacent normal tissues (magnification: ×100 and ×400). (D) RASD2 mRNA levels in paired thyroid cancer and normal tissues measured by qRT‐PCR (*n* = 96 pairs). (E) Kaplan–Meier analysis of 5‐year overall survival in thyroid cancer patients stratified by RASD2 expression (*n* = 96; high vs. low based on median expression). (F) Western blot analysis of RASD2 protein levels across thyroid cell lines: Normal thyroid epithelial cells (Nthy‐ori 3‐1) and thyroid cancer cell lines (BCPAP, IHH4, KTC, and TPC‐1).*p < 0.05, **p < 0.01, ***p < 0.001.

To investigate the clinical relevance of RASD2 in thyroid cancer, we first analyzed RASD2 expression using the GEPIA database, which revealed significant upregulation in thyroid cancer tissues compared to normal controls (Figure 1A). This finding was independently validated using the GEO dataset (GSE197443), showing consistently elevated RASD2 expression across four matched tumor‐normal tissue pairs (Figure 1B). Immunohistochemical staining of a tissue microarray containing 96 paired thyroid cancer specimens and adjacent normal tissues demonstrated markedly increased RASD2 protein levels in tumor tissues (Figure 1C). Consistent with these observations, RT‐qPCR analysis confirmed significantly higher RASD2 mRNA levels in tumor tissues compared to matched normal tissues (Figure 1D). Notably, stratification of patients based on median RASD2 expression revealed that high RASD2 levels significantly correlated with reduced 5‐year overall survival (Figure 1E). Chi‐square analysis demonstrated that elevated RASD2 expression independently predicted poor prognosis and significantly associated with aggressive clinicopathological features, including lymph node metastasis, extrathyroidal extension, and advanced TNM stage, but not with age, gender, or tumor size (Table 1).

### 3.2 RASD2 Silencing Suppresses Thyroid Cancer Cell Growth, Invasion, and Glycolysis

**FIGURE 2:**
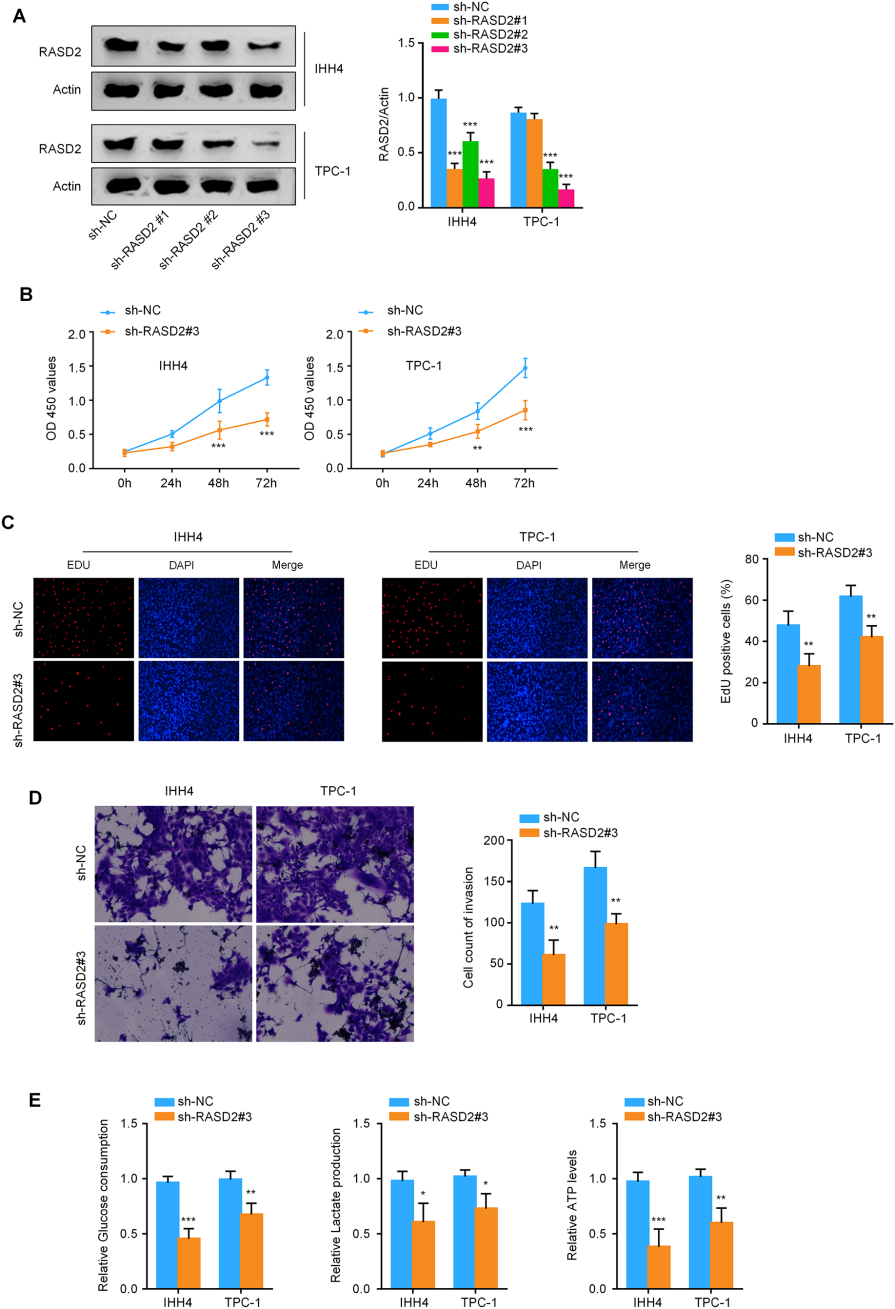
Impact of RASD2 knockdown on thyroid cancer cell behavior. (A) Western blot validation of RASD2 knockdown efficiency using three different shRNAs (sh‐RASD2#1, #2, #3) in IHH4 and TPC‐1 cells. (B) Cell viability assessment by CCK‐8 assay in sh‐RASD2#3 and sh‐NC transfected cells at 24, 48, and 72 h. (C) EdU incorporation assay comparing proliferation between sh‐RASD2#3 and sh‐NC groups. (D) Transwell invasion assay results for sh‐RASD2#3 vs. sh‐NC groups (magnification: ×200). (E) Glycolytic parameters (glucose consumption, lactate production, ATP levels) in sh‐RASD2#3 and sh‐NC transfected IHH4 and TPC‐1 cells. *p < 0.05, **p < 0.01, ***p < 0.001.

To elucidate the biological function of RASD2 in thyroid cancer, we examined RASD2 expression across multiple cell lines. Western blot analysis of thyroid cancer cell lines (BCPAP, IHH4, KTC, and TPC‐1) demonstrated increased RASD2 expression compared to the normal human thyroid epithelial cell line, Nthy‐ori 3‐1. TPC‐1 cells exhibited the highest RASD2 levels, followed by IHH4 cells (Figure 1F). Based on these findings, we selected TPC‐1 and IHH4 cells for functional studies and established stable RASD2 knockdown using lentiviral‐mediated shRNA. Among three tested shRNAs, sh‐RASD2#3 demonstrated the highest knockdown efficiency and was selected for subsequent experiments (Figure 2A). Functional assays revealed that RASD2 knockdown substantially impaired cell viability, as measured by CCK‐8 assay (Figure 2B). EdU incorporation assays showed a marked reduction in proliferating cells following RASD2 silencing compared to control (sh‐NC) groups (Figure 2C). Additionally, Transwell invasion assays demonstrated decreased invasive capacity upon RASD2 knockdown (Figure 2D). To investigate the metabolic impact of RASD2 silencing, we performed glycolytic flux analysis, which revealed reduced glucose consumption, lactate production, and ATP levels in both IHH4 and TPC‐1 cells after RASD2 knockdown (Figure 2E). These findings suggest that RASD2 promotes thyroid cancer progression by modulating cell proliferation, invasion, and glycolytic metabolism.

### 3.3 miR‐485‐5p Directly Targets and Suppresses RASD2 Expression in Thyroid Cancer Cells

**FIGURE 3:**
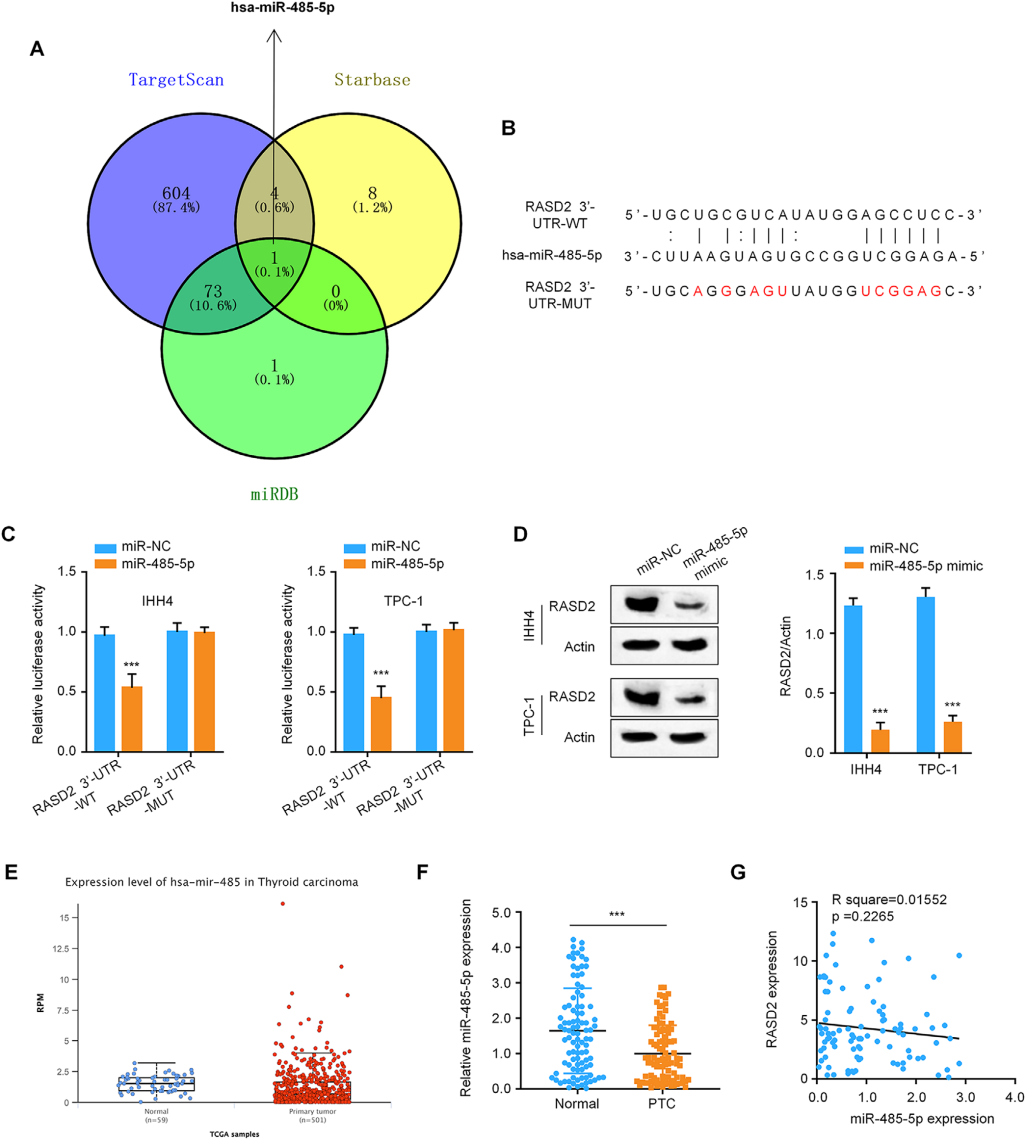
miR‐485‐5p regulation of RASD2 in thyroid cancer. (A) Venn diagram showing overlapping miRNA predictions from three databases (TargetScan, miRDB, StarBase). (B) Sequence alignment between miR‐485‐5p and RASD2 3′‐UTR showing predicted binding site. (C) Dual‐luciferase reporter assay results in IHH4 and TPC‐1 cells transfected with wild‐type or mutant RASD2 3′‐UTR constructs and miR‐485‐5p mimic or negative control. (D) Western blot analysis of RASD2 protein levels in cells transfected with miR‐485‐5p mimic or negative control. (E) TCGA database analysis of miR‐485‐5p expression in thyroid cancer vs. normal tissues. (F) miR‐485‐5p expression in paired tumor‐normal tissues by qRT‐PCR (*n* = 96 pairs). (G) Correlation analysis between miR‐485‐5p and RASD2 expression levels in thyroid cancer tissues. *p < 0.05, **p < 0.01, ***p < 0.001.

Bioinformatic analysis using three independent miRNA target prediction platforms (miRDB, StarBase, and Targetscan) identified potential miRNAs targeting RASD2. Through comparative analysis of these predictions, hsa‐miR‐485‐5p emerged as the only miRNA consistently predicted by all three algorithms (Figure 3A). Sequence alignment revealed precise complementarity between the miR‐485‐5p seed region and a conserved site within the RASD2 3′′‐UTR (Figure 3B). To validate this predicted interaction, we performed dual‐luciferase reporter assays, which demonstrated that miR‐485‐5p overexpression suppressed luciferase activity in cells expressing wild‐type RASD2 3′‐UTR constructs. This repressive effect was abolished when the predicted binding site was mutated, confirming RASD2 as a direct target of miR‐485‐5p (Figure 3C). Western blot analysis showed that transfection of miR‐485‐5p mimics led to marked reduction of RASD2 protein levels in both IHH4 and TPC‐1 cells (Figure 3D). Analysis of TCGA data revealed consistent downregulation of miR‐485‐5p in thyroid cancer specimens compared to normal tissues (Figure 3E). This finding was further validated through qRT‐PCR analysis of our cohort of 96 paired thyroid cancer tissues (Figure 3F). Correlation analysis demonstrated an inverse relationship between miR‐485‐5p and RASD2 expression levels in thyroid cancer tissues (Figure 3G). Collectively, these findings establish miR‐485‐5p as a negative regulator of RASD2 in thyroid cancer, suggesting a potential tumor‐suppressive role for this miRNA.

### 3.4 MiR‐485‐5p Suppresses Thyroid Cancer Progression Through RASD2 Inhibition

**FIGURE 4:**
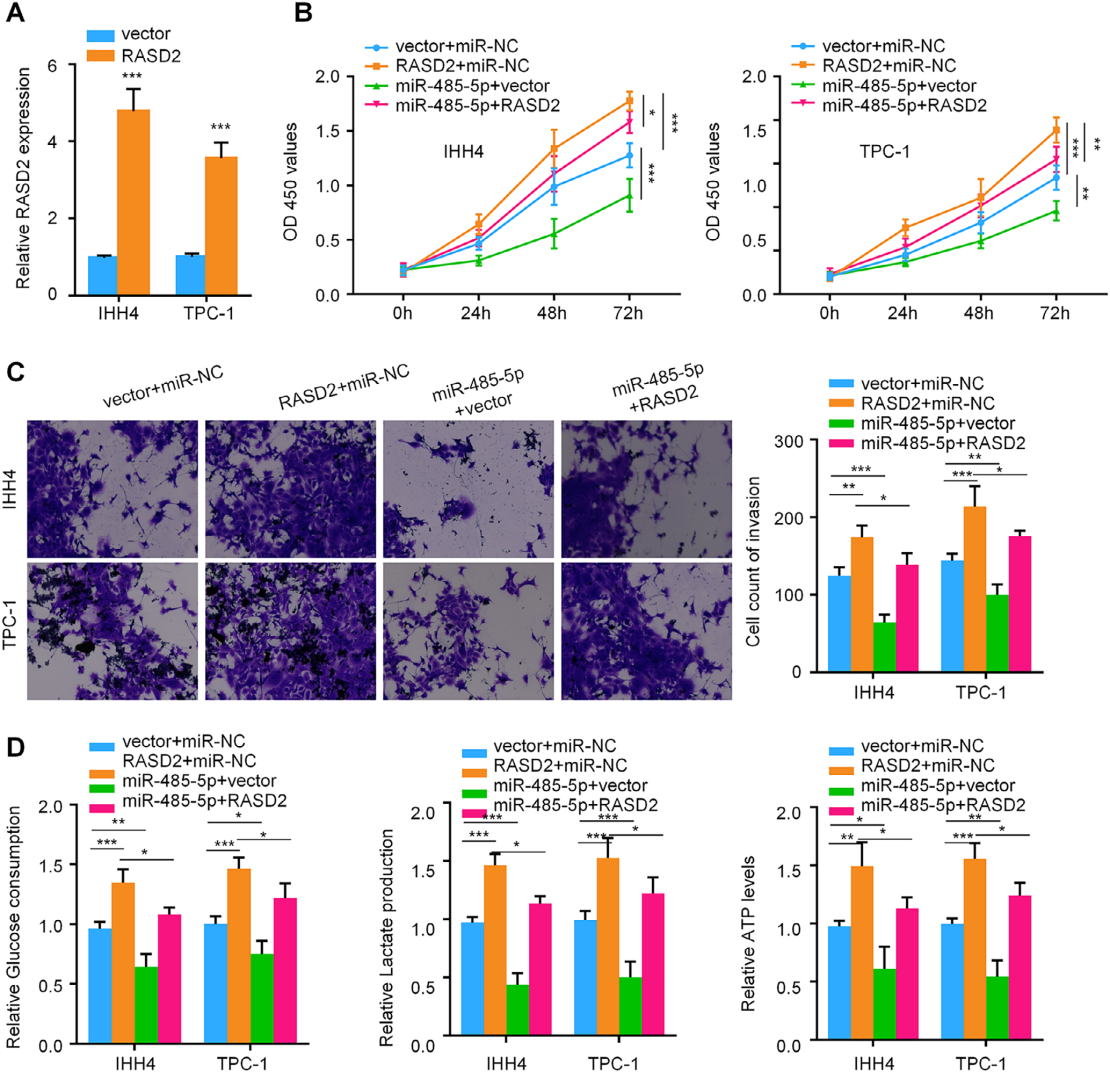
Functional interaction between miR‐485‐5p and RASD2. (A) RT‐PCR validation of RASD2 overexpression in IHH4 and TPC‐1 cells. (B) CCK‐8 proliferation assay comparing four groups: Vector + miR‐NC, RASD2 + miR‐NC, vector + miR‐485‐5p, and RASD2 + miR‐485‐5p. (C) Transwell invasion assay results across all four experimental groups. (D) Analysis of glycolytic parameters (glucose consumption, lactate production, ATP levels) in cells under different treatment conditions. *p < 0.05, **p < 0.01, ***p < 0.001.

To establish the functional relationship between miR‐485‐5p and RASD2, we conducted comprehensive rescue experiments. Following validation of RASD2 overexpression efficiency by qRT‐PCR (Figure 4A), we examined cellular phenotypes under various conditions. CCK‐8 proliferation assays revealed that while RASD2 overexpression enhanced cell growth, miR‐485‐5p overexpression exhibited growth‐suppressive effects. Notably, co‐expression of RASD2 with miR‐485‐5p partially reversed the anti‐proliferative effect of miR‐485‐5p (Figure 4B). Similarly, Transwell invasion assays demonstrated that RASD2 overexpression promoted cell invasion, whereas miR‐485‐5p overexpression reduced invasive capacity. Co‐transfection of RASD2 with miR‐485‐5p partially restored the invasive phenotype (Figure 4C). Analysis of cellular metabolism showed that RASD2 overexpression enhanced glucose consumption, lactate production, and ATP levels compared to controls, while miR‐485‐5p overexpression suppressed these glycolytic parameters. The inhibitory effects of miR‐485‐5p on glycolysis were partially rescued by RASD2 co‐expression (Figure 4D). These results demonstrate that miR‐485‐5p exerts its tumor‐suppressive functions primarily through downregulation of RASD2, establishing a novel regulatory axis in thyroid cancer progression.

### 3.5 RASD2 Knockdown Suppresses Thyroid Cancer Growth and Metastasis in Xenograft Models

**FIGURE 5:**
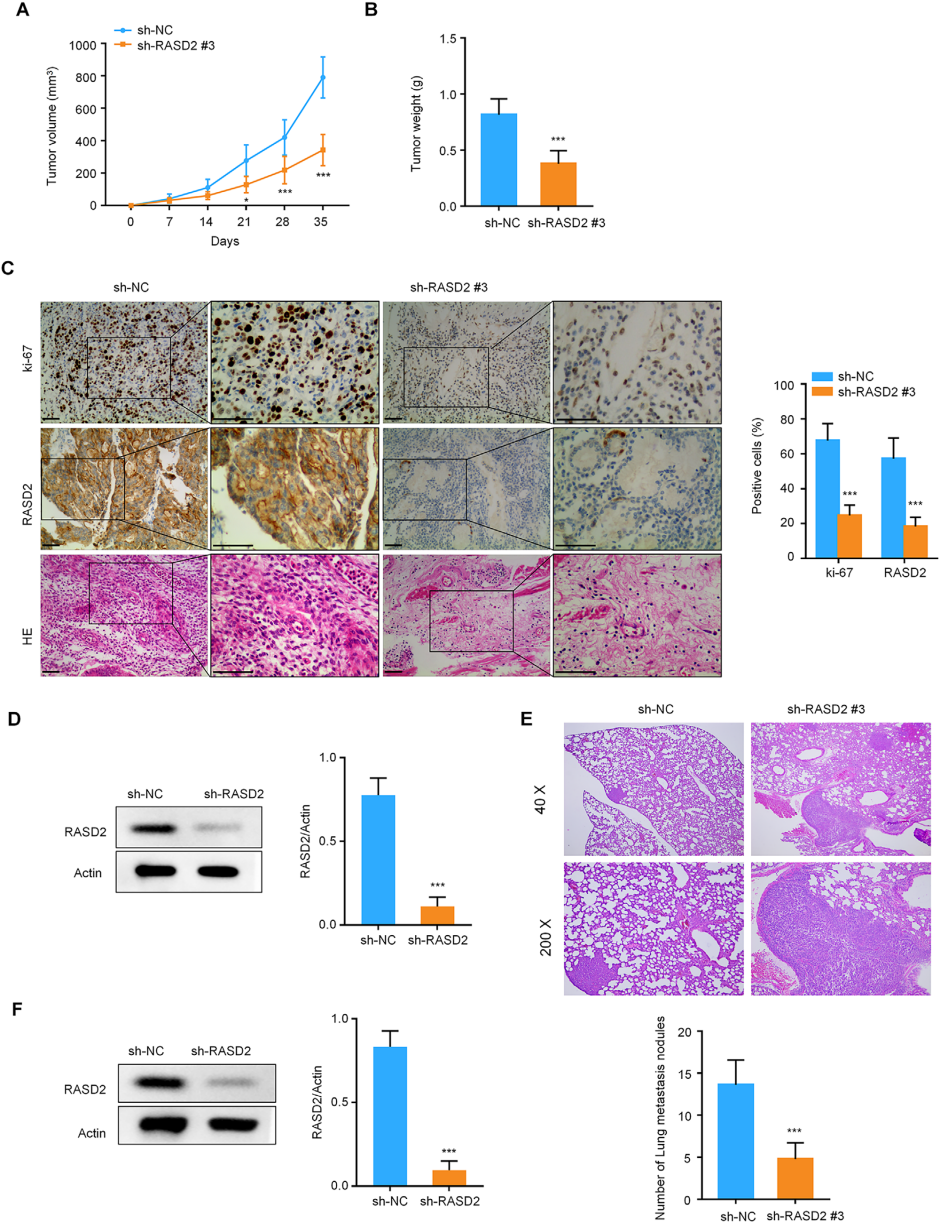
In vivo effects of RASD2 knockdown. (A) Tumor growth curves from subcutaneous xenografts of TPC‐1 cells (sh‐RASD2#3 vs. sh‐NC; *n* = 5 mice/group) measured weekly for 5 weeks. (B) Final tumor weights at study endpoint (day 35). (C) H&E staining and immunohistochemical analysis of Ki‐67 and RASD2 in xenograft tissues (magnification: ×200 and ×400). (D) Immunoblotting analysis of RASD2 expression in tumor samples. (E) Representative H&E staining of lung sections from experimental metastasis model (magnification: ×40 and ×200; *n* = 5 mice/group). Quantification of lung metastatic foci in sh‐RASD2#3 vs. sh‐NC groups. (F) Immunoblotting analysis of RASD2 expression in metastatic tumor samples. Compared to the sh‐NC group, *p < 0.05, **p < 0.01, ***p < 0.001.

To validate our in vitro findings in a physiological context, we established subcutaneous xenograft models using TPC‐1 cells with stable RASD2 knockdown. Monitoring of tumor development revealed that RASD2 silencing substantially reduced both tumor growth rates and final tumor masses compared to sh‐NC controls (Figure 5A,B). Immunohistochemical and immunoblotting examination of harvested tumor tissues demonstrated markedly decreased Ki‐67 proliferation marker and RASD2 expression in the knockdown group (Figure 5C,D). To investigate the role of RASD2 in metastatic spread, we employed an experimental lung metastasis model using tail vein injection of TPC‐1 cells. Animals receiving sh‐RASD2‐transfected cells developed fewer lung metastases compared to those injected with sh‐NC control cells, with confirmed RASD2 downregulation in sh‐RASD2 tumor samples (Figure 5E,F). These in vivo results provide compelling evidence that RASD2 plays a crucial role in promoting both primary tumor growth and metastatic colonization in thyroid cancer.

## 4 Discussion

Thyroid cancer incidence has risen dramatically worldwide, emerging as one of the most rapidly increasing malignancies of the endocrine system. According to GLOBOCAN 2020 statistics, thyroid cancer accounted for 586,000 new cases and 43,600 deaths globally [16, 17]. PTC represents the predominant histological subtype, comprising over 80% of all thyroid cancer diagnoses [18]. While thyroid cancer generally exhibits favorable outcomes with a relatively low mortality rate of 5% [19]. current therapeutic strategies face significant challenges in managing advanced disease. The high rates of lymph node metastasis (50%) and disease recurrence (20%) underscore the urgent need for more effective treatment approaches [20]. These clinical challenges emphasize the critical importance of elucidating the molecular mechanisms underlying thyroid cancer progression and metastasis to develop novel therapeutic strategies.

While established risk factors for PTC include exposure to ionizing radiation, dysregulated iodine intake, and genetic susceptibility [21], the molecular mechanisms driving tumor initiation and progression remain incompletely understood. Our study provides novel insights into PTC pathogenesis by identifying RASD2 as a key regulatory molecule. Through integrated analysis of GEO and TCGA datasets, we demonstrated significant upregulation of RASD2 in PTC tissues compared to normal thyroid tissue. Importantly, elevated RASD2 expression strongly correlated with adverse clinical outcomes, suggesting its potential value as a prognostic biomarker. These initial observations led us to investigate the molecular mechanisms through which RASD2 promotes thyroid cancer progression, focusing particularly on its role in tumor growth, metastasis, and metabolic reprogramming.

RASD2, a member of the Ras superfamily of small GTPases, was initially characterized as an mTOR1 signaling pathway activator with diverse physiological functions, including myelin formation, axonal growth, and neurotransmitter regulation through hippocampal dopamine D2 receptors [22, 23, 24]. While early studies focused on its neurological roles, the oncogenic potential of RASD2 was first revealed by Gromova et al. [7], who identified its aberrant upregulation in gastrointestinal tumors. Subsequent investigations have expanded RASD2's oncogenic portfolio, demonstrating its crucial role in tumor immune evasion in gliomas [5]. and promotion of proliferation and metastasis through enhanced glycolysis in melanoma [8]. Of particular relevance to our study, research in Parkinson's disease unexpectedly revealed RASD2's influence on thyroid hormone homeostasis [25], suggesting potential endocrine regulatory functions. Building on these observations, our investigation provides the first comprehensive characterization of RASD2's role in thyroid cancer through complementary in vitro, in vivo, and clinical analyses. Through integrated analysis of published datasets, coupled with extensive cellular studies across multiple thyroid cancer cell lines, we demonstrated consistent RASD2 upregulation in both primary thyroid cancer tissues and malignant cell lines compared to their normal counterparts. The functional significance of this upregulation was confirmed through loss‐of‐function studies, where RASD2 knockdown significantly impaired tumor cell proliferation and invasion, and attenuated both tumor growth and metastatic potential in xenograft models. Importantly, elevated RASD2 expression strongly correlated with aggressive clinicopathological features, including lymph node metastasis, extrathyroidal extension, and advanced TNM staging. These findings indicate that RASD2 may serve as an independent predictor of poor prognosis and a potential therapeutic target.

Metabolic reprogramming, particularly the Warburg effect, represents a fundamental hallmark of cancer cells, characterized by preferential utilization of glycolysis for energy production even under aerobic conditions [26]. This metabolic shift provides cancer cells with not only rapid ATP generation but also essential biosynthetic intermediates required for sustained proliferation, invasion, and angiogenesis [27]. Thyroid cancer cells exhibit particularly pronounced dependence on glycolytic metabolism, suggesting its crucial role in disease progression [28]. Our investigation revealed that RASD2 functions as a key regulator of this metabolic phenotype in thyroid cancer. Specifically, RASD2 overexpression significantly enhanced glycolytic flux, as evidenced by increased glucose consumption, lactate production, and ATP generation. Conversely, RASD2 silencing markedly attenuated these glycolytic parameters, concurrent with reduced proliferative and invasive capabilities. These findings establish glycolytic reprogramming as a major mechanism through which RASD2 promotes thyroid cancer progression, offering potential therapeutic implications through metabolic targeting.

MicroRNAs (miRNAs) serve as critical post‐transcriptional regulators by targeting specific recognition sites within the 3′‐UTR of messenger RNAs [29, 30]. In thyroid cancer, these molecular regulators orchestrate complex networks controlling cell proliferation, differentiation, and survival [31, 32]. While the miR‐200 family has been shown to regulate RASD2 in podocyte differentiation [33], our investigation identifies miR‐485‐5p as a novel upstream regulator of RASD2 in thyroid cancer. Although miR‐485‐5p dysregulation has been documented in various malignancies, including ovarian cancer, osteosarcoma, and colorectal cancers [14, 34, 35, 36, 37], its role in thyroid cancer remained unexplored. Our luciferase reporter assays confirmed direct binding of miR‐485‐5p to the predicted binding site in RASD2′s 3′UTR. When this binding site was mutated, the regulatory effect was lost, confirming the specificity of this interaction. The binding of miR‐485‐5p to RASD2 mRNA likely reduces RASD2 expression through two classical miRNA mechanisms: either by promoting mRNA degradation (reducing stability) or by inhibiting translation. This direct targeting was further validated by the observation that miR‐485‐5p overexpression decreased RASD2 protein levels. These molecular findings explain how miR‐485‐5p functions as a tumor suppressor in thyroid cancer through RASD2 regulation. From a clinical perspective, these findings suggest that targeting the miR‐485‐5p/RASD2 axis could represent a promising therapeutic strategy. However, whether miR‐485‐5p can serve as a reliable biomarker for early diagnosis of thyroid cancer requires further clarification through large‐scale clinical validation studies.

Several limitations of our study warrant further investigation. While we demonstrate that RASD2 significantly influences glycolytic metabolism in thyroid cancer, the precise molecular mechanisms underlying this regulation remain to be fully elucidated. Future studies should explore potential interactions between RASD2 and key metabolic enzymes or signaling pathways, such as HIF‐1α, c‐Myc, or mTOR, which might mediate its effects on glycolysis. Additionally, although we establish miR‐485‐5p as a direct regulator of RASD2, other potential upstream regulators may contribute to RASD2 dysregulation in thyroid cancer. Furthermore, our findings would benefit from validation in larger patient cohorts and diverse thyroid cancer subtypes. While we focused on RASD2 as a target of miRNA‐485‐5p, future studies should investigate other downstream targets of miRNA‐485‐5p that may also contribute to thyroid cancer progression, providing a more complete understanding of miRNA‐485‐5p's role in this disease. Furthermore, comparative studies between RASD2 silencing and established glycolysis inhibitors would help position RASD2‐targeted therapy within the broader landscape of metabolic interventions in thyroid cancer. The complex signaling network downstream of RASD2 requires further investigation to identify potential therapeutic targets and resistance mechanisms. Moreover, while miR‐485‐5p restoration shows promise as a therapeutic strategy, its clinical feasibility needs careful evaluation considering potential off‐target effects and the challenges of miRNA‐based therapeutics delivery.

To conclude, our study identifies RASD2 as a crucial driver of thyroid cancer progression and a potential prognostic biomarker. Through systematic investigation, we uncover a novel regulatory axis wherein miR‐485‐5p suppresses RASD2 expression, thereby modulating glycolytic metabolism and malignant behavior. Notably, this miR‐485‐5p/RASD2 axis is dysregulated in thyroid cancer, with diminished miR‐485‐5p expression leading to RASD2 upregulation. These findings not only provide mechanistic insights into thyroid cancer metabolism but also identify promising therapeutic targets for intervention strategies.

## Ethics Statement

The investigation adhered to the ethical principles outlined in the Declaration of Helsinki (Code of Ethics, AF‐SOP‐07‐1.1‐01) and received approval from the The Second Hospital of Hebei Medical University Ethics Committee. Each animal experimental procedure gained approval from Animal Ethics Committee of The Second Hospital of Hebei Medical University. The experimental protocol was performed in accordance with the relevant guidelines and regulations of the Basel Declaration. The study is reported in accordance with ARRIVE guidelines (https://arriveguidelines.org).

## Conflicts of Interest

The authors declare no conflicts of interest.

## Data Availability

The datasets used and/or analyzed during the current study are available from the corresponding author via email request.
